# Intestinal Bacteria Condition Dendritic Cells to Promote IgA Production

**DOI:** 10.1371/journal.pone.0002588

**Published:** 2008-07-02

**Authors:** Joanna C. Massacand, Patrick Kaiser, Bettina Ernst, Aubry Tardivel, Kurt Bürki, Pascal Schneider, Nicola L. Harris

**Affiliations:** 1 Environmental Biomedicine, Institute of Integrative Biology, Swiss Federal Institute of Technology, Zürich-Schlieren, Switzerland; 2 Molecular Biomedicine, Institute of Integrative Biology, Swiss Federal Institute of Technology, Zürich-Schlieren, Switzerland; 3 Department of Biochemistry, University of Lausanne, Epalinges, Switzerland; 4 Institute of Laboratory Animal Science, University of Zürich, Zürich, Switzerland; Vanderbilt University School of Medicine, United States of America

## Abstract

Immunoglobulin (Ig) A represents the predominant antibody isotype produced at the intestinal mucosa, where it plays an important role in limiting the penetration of commensal intestinal bacteria and opportunistic pathogens. We show in mice that Peyer's Patch-derived dendritic cells (PP-DC) exhibit a specialized phenotype allowing the promotion of IgA production by B2 cells. This phenotype included increased expression of the retinaldehyde dehydrogenase 1 (RALDH1), inducible nitric oxide synthase (iNOS), B cell activating factor of the tumor necrosis family (BAFF), a proliferation-inducing ligand (APRIL), and receptors for the neuropeptide vasoactive intestinal peptide (VIP). The ability of PP-DC to promote anti-CD40 dependent IgA was partially dependent on retinoic acid (RA) and transforming growth factor (TGF)-β, whilst BAFF and APRIL signaling were not required. Signals delivered by BAFF and APRIL were crucial for CD40 independent IgA production, although the contribution of B2 cells to this pathway was minimal. The unique ability of PP-DC to instruct naïve B cells to differentiate into IgA producing plasma cells was mainly imparted by the presence of intestinal commensal bacteria, and could be mimicked by the addition of LPS to the culture. These data indicate that exposure to pathogen-associated molecular patterns present on intestinal commensal bacteria condition DC to express a unique molecular footprint that in turn allows them to promote IgA production.

## Introduction

The intestinal immune response is highly specialized towards IgA production, with up to 3 g of secretory IgA (SIgA) secreted into the human intestinal lumen per day [1], [2]. SIgA functions to provide a barrier against the penetration of intestinal commensal bacteria and invasive pathogens [3], [4], however the exact mechanisms regulating the selective production of this antibody isotype at mucosal sites remain unknown. PP are the major site of intestinal B cell IgA class-switch recombination, with switched cells leaving this organ and migrating via the mesenteric lymph nodes to the thoracic duct to finally enter the intestinal lamina propria. Here they differentiate into plasma cells secreting dimeric IgA linked by the J chain [5]. Dimeric IgA associates with the J chain to form SIgA, which binds the polymeric Ig receptor (pIgR) expressed basolaterally on epithelial cells [6], [7] and is transported across the epithelium to be released into the intestinal lumen. A large component of circulating plasma IgA present in mice is also derived from the intestinal mucosa, and can be transported to the intestinal lumen via the hepatobillary pathway [8].

The large majority of lymphoid derived B cells are B2 cells, and it was believed for a long time that the induction of IgA class-switch recombination in these cells required cognate T cell help [9], [10] together with the presence of cytokines such as TGF-β [11]. However, we now know that many other factors can regulate antibody isotype switching including DC-expressed BAFF (also known as BLys) and APRIL [12], or direct B cell ligation by Toll-like receptor ligands [13], [14]. Moreover, IgA production has been described in mice which lack CD4^+^ T cells [15], [16] or are unable to support MHC II-dependent T-B cell cognate interactions [17]. T cell-independent IgA production is thought to be mainly derived from B1 cells [15], [16], whilst the degree to which B2 cells contribute to this pathway remains unclear [18].

Early landmark studies have shown that in addition to promoting IgA, intestinal antigens selectively promote the activation of T and B lymphocytes that upregulate α4β7 integrin on their surface which allows their migration to the intestinal lamina propria [19]–[21]. We are only now beginning to unravel the complexity of how this process is regulated, with a wealth of new data implicating a central role for DC from gut-associated lymphoid tissues (GALT-DC) [22]–[26]. DC typically function to regulate adaptive T cell responses, although an increasing number of reports indicate that they can additionally regulate B cell responses [27]–[30]. These studies raise the question as to how GALT-DC are imparted with a specialized intestinal phenotype, and indicate that these cells may in fact impact on intestinal B cell responses.

We show that PP-DC express a specialized molecular footprint allowing these cells to preferentially promote the differentiation of naïve B2 cells into IgA producing plasma cells. Furthermore, we demonstrate that PP-DC are conditioned to promote IgA production in conventional mice by the presence of commensal intestinal bacteria.

## Materials and Methods

### Mice

C57BL/6 were purchased from Charles River Laboratories Inc. and housed at Biosupport AG under specific pathogen free (SPF) conditions in ventilated filter top cages. Germ-free (GF) C57BL/6 mice were bred under gnotobiotic conditions at the Institute of Laboratory Animal Science of the University of Zürich. Mice doubly deficient for TACI (transmembrane activator and calcium-modulator and cyclophilin ligand interactor) and BCMA (B-cell maturation antibody) (TACIxBCMA^o/o^) were generated at, and kindly provided by, Biogen Inc (Cambridge, MA)[31]. All animal experiments were performed according to institutional guidelines and to Swiss federal and cantonal laws on animal protection.

### DC and B cell isolation

Animals were sacrificed by CO_2_ inhalation and subsequent cervical dislocation and DC isolated from the PP or peripheral lymph nodes (PLN) of naïve mice. For this purpose lymphoid tissues were digested twice for 30 min at 37°C in DC medium (IMDM (BioWhittaker) containing 7% FCS, L-glutamine, Hepes, 100 U/ml penicillin and 100 µg/ml streptomycin (Gibco)) containing 10 U/ml collagenase IV (Worthington Biochemical Corporation). At the end of each incubation period, tissue fragments were harvested and passed through a 40 µm cell strainer (BD biosciences). CD11c positive cells were then positively selected using anti-CD11c labelled MicroBeads (Miltenyi Biotec) according to the manufacturers instructions. The purity of positively selected cells was assessed by FACS analysis and was approximately 70% for all DC populations. For all experiments shown data was reproduced at least once using DC further purified to greater than 95% purity by sorting using a FACSVantage®.

B cells were isolated from the spleen of naïve mice by incubation of cell suspensions with FITC-labelled anti-mouse IgD^b^ (BD Pharmingen), and positive selection using anti-FITC-coated MicroBeads (Miltenyi Biotec). Naïve B cell preparations were always found to be greater than 96% pure.

To obtain peritoneal cells, naïve C57BL/6 mice were sacrificed and the peritoneal cavity was gently washed with 10 ml sterile PBS. Peritoneal B cells were detected based on their CD19 expression. B-1 and B-2 B cells were differentiated by the IgD, IgM and CD5 expression of CD19^+^ B cells.

### Flow cytometry and antibodies

The following antibodies were used to assess DC and B cell purity and activation phenotypes and were purchased from eBioscience: FITC-labelled anti-mouse CD11c, PE-labelled anti-mouse CD11b, biotinylated anti-mouse MHCII, biotinylated anti-mouse CD80, APC-labelled anti-mouse CD19, PE-labelled anti-mouse TCRβ, PE-labelled anti-mouse IgM, biotinylated anti-mouse CD5, APC-labelled streptavidin and PE-labelled streptavidin. Live cells were gated based on propidium iodide (BD Pharmingen) exclusion during acquisition on a FACSCalibur® (Becton Dickinson). Flow cytometric analysis was performed using FlowJo software (TreeStar, Inc.).

### DC-B co-cultures

CD11c^+^ DC were cultured together with IgD^+^ B cells at a 1∶5 ratio in round bottom 96-well plates (Corning). Cells were either cultured with complete medium only (DC medium containing 25 µM 2-ME) or with additional anti-CD40 (clone FGK-45, provided by Cytos Biotechnology) at 5 or 2 µg/ml as specified. LPS (1 µg/ml, cell culture LPS from *Escherichia coli* 0111:B4, Sigma-Aldrich) was added to some cultures as a positive control.

In some experiments, the RA receptor β (RARβ) inhibitor LE135 (Tocris Bioscience) and monoclonal anti-TGF-β (Sigma-Aldrich) were added to PP-DC-B cell co-cultures at 1 µM and 10 µg/ml respectively.

Duplicates or triplicates were prepared for each set of culture conditions. Cells were cultured for 7 days at 37°C and 5% CO_2_ after which the supernatant was collected and frozen at −20°C for later analysis.

### Real-time quantitative RT-PCR

Real-time quantitative RT-PCR was performed using cDNA isolated from CD11c^+^ PP- or PLN-DC isolated from naïve SPF mice. cDNA was prepared from total RNA isolated using TRI Reagent (Molecular Research Center, Inc.), treated with DNase (Invitrogen) to avoid genomic DNA contamination, and reverse transcribed using the SuperScript III RT kit (Invitrogen). Quantitative real-time RT-PCR was performed using Brilliant SYBR Green (Stratagene) in an iCycler (Bio-Rad Laboratories). Expression was normalized to the housekeeping gene GAPDH. Sequences of all primers used were: GAPDH (Gapdh) 5′-GGG TGT GAA CCA CGA GAA AT-3′ and 5′-CCT TCC ACA ATG CCA AAG TT-3′; BAFF (Tnfsf13b) 5′-AGG CTG GAA GAA GGA GAT GAG-3′ and 5′-CAG AGA AGA CGA GGG AAG GG-3′; APRIL (Tnfsf13) 5′-GGG GAA GGA GTG TCA GAG TG-3′ and 5′-GCA GGG AGG GTG GGA ATA C-3′; TGF-β (Tgfb1) 5′-TGG AGC AAC ATG TGG AAC TC-3′ and 5′-TGC CGT ACA ACT CCA GTG AC-3′; VPAC1 (Vipr1) 5′-CTC ATC CCT CTG TTC GGA GTT C-3′ and 5′-CGA CGA GTT CGA AGA CCA TTT T-3′; VPAC2 (Vipr2) 5′-GGA CAG CAA CTC GCC TCT CT-3′ and 5′-AGA ATG GGC ATC CGA ATG AC-3′; RALDH1 (Aldh1a1) 5′-ATG GTT TAG CAG CAG GAC TCT TC-3′ and 5′-CCA GAC ATC TTG AAT CCA CCG AA-3′; iNOS (Nos2) 5′-CTG CCT CAT GCC ATT GAG TT-3′ and 5′-TGA GCT GGT AGG TTC CTG TTG-3′.

### Detection of antibody isotypes by ELISA

Harvested culture supernatants were analysed for IgA, IgG1 or IgM antibody isotype levels using standard ELISA assays. In brief, 96 well plates (Maxisorp; Nunc) were coated with unlabeled goat anti-mouse antibodies to either IgA, IgG1 or IgM (Southern Biotech) in ELISA coating buffer (5.88 g/l NaHCO_3_, 3.18 g/l Na_2_CO_3_ (Fluka) in ddH_2_O, pH 9.6) overnight at 4°C. Plates were washed with PBS/0.05% Tween®20 (Fluka) and blocked with PBS/1% BSA for 2 h at room temperature (RT). Supernatants were serially diluted in PBS/0.1% BSA starting with a 1.5 fold dilution for IgA and IgG1 detection, and 3 fold dilution for IgM. Purified mouse IgA (BD Pharmingen), mouse IgG1 or mouse IgM (both from Southern Biotech) were used as standards and were serially diluted in parallel to the samples starting from a concentration of 3 µg/ml and according to the isotype of the coating antibody. Samples and standards were incubated for 2 h at RT then washed extensively and incubated with alkaline-phosphatase-labeled goat anti-mouse antibodies to IgA, IgG1 or IgM (Southern Biotech) diluted into PBS/0.1% BSA, according to the isotype of the coating antibody. Plates were incubated for a further 1 h at RT, washed extensively then developed using the substrate p-nitrophenyl phosphate (Sigma-Aldrich). ODs were measured on an ELISA reader (Bucher Biotec) at 405 nm.

### Statistical analysis

For all IgA data, significant differences, between the indicated group and the ‘no DC control cultures’, were determined by a one-tailed student's t-test. In instances where the ‘no DC culture’ gave a value below the detection limit, the sample was given a value of 1.0 ng/ml which represents the detection limit of the IgA ELISA. In experiments where the inhibitory effect of LE135 and anti-TGF-β was studied, the significant differences between the reagents as compared to medium was determined by a one-tailed student's t-test. In all cases p values are depicted as *p≤0.05, **p≤0.005 or ***p≤0.0005.

## Results

### PP-DC promote IgA production

**Figure 1:**
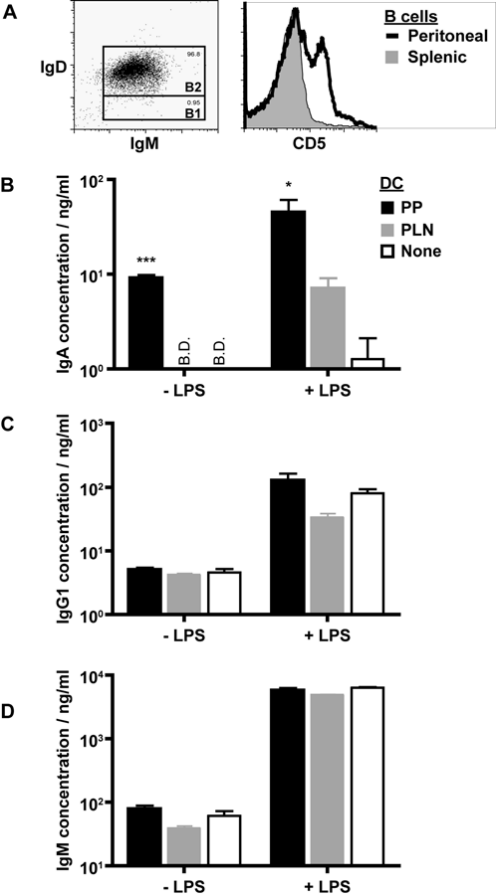
PP-DC promote IgA production by B cells. (A) The dot plot (left panel) shows IgM and IgD expression by MACS sorted splenic IgD^+^ B cells. Gates depict putative B2 and B1 cell populations. The histogram plot (right panel) shows CD5 expression by splenic IgD^+^ B cells (shaded area) or peritoneal B cells (solid line) gated for CD19 expression. (B–D) Splenic IgD^+^ B cells from naïve C57BL6 mice were cultured alone (open bars) or together with CD11c^+^ DC isolated from the PP (black bars) or PLN (grey bars). Anti-CD40 mAb (2 µg/ml) was added to all cultures in the absence or presence of additional LPS (1 µg/ml) as indicated. At the end of 7 days of culture, supernatants were collected and (B) IgA, (C) IgG1 and (D) IgM antibody concentrations determined by standard ELISA assay. Cultures were performed in triplicate and the mean±S.E.M. are shown. B.D. depicts those samples where the antibody concentration was below the detection limit of the ELISA assay. No IgA was detected in control cultures containing DC alone. The data shown are from one experiment and are representative of at least three independent experiments.

To investigate the direct impact of DC on B cell antibody class switching we established an *in vitro* co-culture assay whereby purified DC were incubated together with naïve IgD^+^ B cells. B cells were isolated from the spleen and were found to be 96% IgM^hi^IgD^hi^ representing a B2 cell phenotype (Figure 1A). Less than 1% of the cells expressed a B1 cell phenotype (IgM^hi^IgD^lo^), as confirmed by the lack of CD5 expression (Figure 1A). T cell help was substituted in these cultures by the addition of stimulatory anti-CD40 monoclonal antibody such that T cell-derived cytokines did not influence the experimental outcome. Culture supernatant was harvested after 7 days of culture and the production of IgA, IgG1 and IgM antibody isotypes analyzed by standard ELISA assays. Addition of DC to naïve B cells did not impact on CD40-dependent IgM or IgG1 production and LPS substantially increased the production of both isotypes (Figure 1C&D). In contrast, DC were required for IgA production either in the absence or presence of additional LPS (Figure 1B). The ability of DC to promote CD40-dependent IgA production was most striking when the DC were isolated from the PP with PP-DC being significantly (p = 0.0004) better than PLN-DC at promoting IgA production. Addition of LPS to the culture rendered all DCs capable of promoting IgA production (in this case no significant difference was noted between PP- and PLN-DC, although the addition of PP-DC resulted in higher yields of IgA)(Figure 1B). These data indicate that DC can impact directly on naïve B2 cells to promote CD40-dependent IgA production and that this effect is most dramatic when DC are isolated from the PP.

### PP-DC exhibit a specialized molecular phenotype

**Figure 2:**
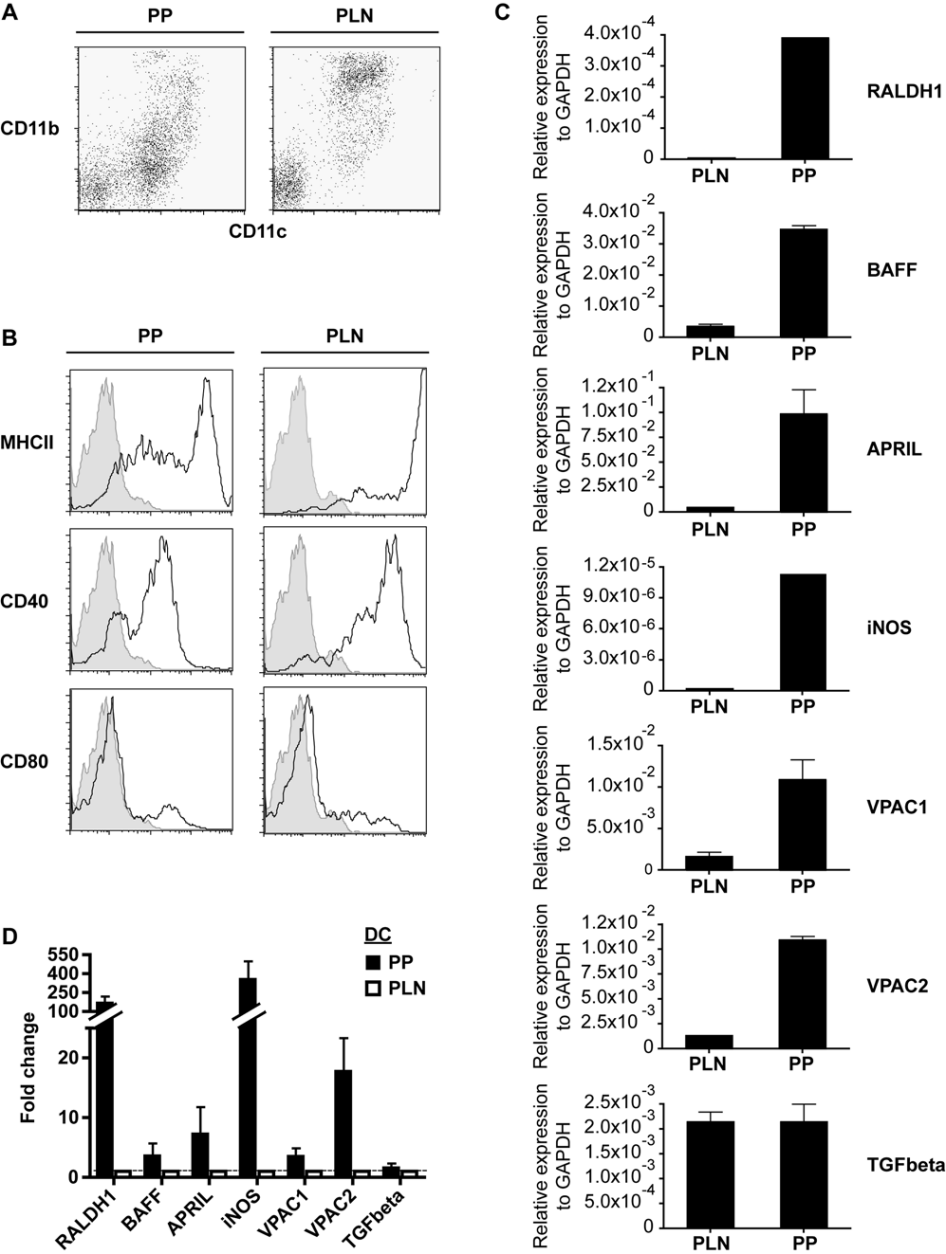
PP-DC express a distinct molecular footprint. (A) Dot plots show MACS sorted CD11c^+^ DC isolated from the PP or PLN and stained with antibodies against CD11c and CD11b. (B) Histrogram plots show cells from (A) gated for CD11c expression and stained with antibodies against MHC class II, CD40 or CD80 as indicated. All plots show expression levels of the indicated activation marker (solid line) as compared to unstained CD11c^+^ control populations (shaded area). All data shown are from one experiment and are representative of at least three independent experiments. (C) Relative mRNA levels to GAPDH for the indicated genes as determined for CD11c^+^ DC isolated from the PP or PLN by quantitative real time PCR. Results shown are from representative measurements. (D) Same as (C) shown as fold change for PP-DC (black bars) relative to PLN-DC (open bars) for which mRNA expression was normalized to a value of 1. Data represent the mean±S.E.M. of combined values from at least three independent experiments.

Numerous factors have been demonstrated to promote IgA production including BAFF and APRIL [12], the neuropeptide VIP [32], [33], TGF-β [11], RA [25] and iNOS [34]. Since PP-DC were more potent at driving IgA production than their PLN counterparts we compared their expression of these factors. DC isolated from the PP were found to represent distinct subpopulations based on their CD11c and CD11b surface expression and exhibited an overall less activated phenotype than those isolated from the PLN (Figure 2A&B). In keeping with the observations of others [22] PP-DC expressed increased levels of mRNA encoding for RALDH1 which is required for RA production (Figure 2C&D). PP-DC also exhibited increased levels of BAFF, APRIL, iNOS and VIP receptors (VPAC1 and VPAC2) mRNA as compared to PLN-DC (Figure 2C&D). We did not note any difference between PP-DC or PLN-DC for TGF-β expression (Figure 2C&D). However, TGF-β is secreted as a latent molecule requiring activation by tissue plasminogen activator and tissue plasminogen activator has been demonstrated to be expressed on CD103^+^ DC from the mesenteric lymph nodes raising the possibility that PP-DC may also result in the generation of higher amounts of active TGF-β [35].

**Figure 3:**
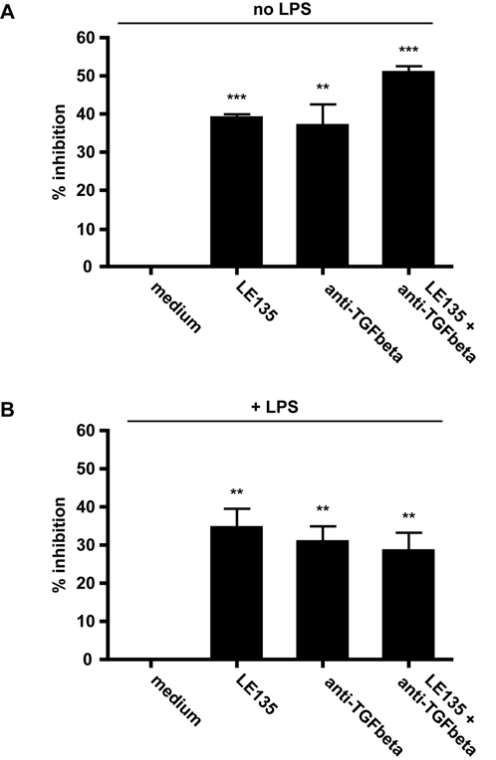
RA and TGF-β are involved in PP-DC mediated IgA production. Naïve splenic IgD^+^ B cells from C57BL/6 mice were cultured together with CD11c^+^ DC from the PP. Anti-CD40 mAb (5 µg/ml) was added to all cultures in the absence (A) or presence (B) of additional LPS (1 µg/ml). LE135, anti-TGF-β or the combination of both were added where indicated. At the end of 7 days of culture, supernatants were collected and IgA concentration determined by standard ELISA assay. The inhibitory effect of each reagent and their combination was calculated by comparing their IgA concentrations to control cultures containing medium alone, and are depicted as percent inhibition, medium being equivalent to 0% inhibition. Cultures were performed in triplicate and the mean±S.E.M. are shown. The data shown are from one experiment and are representative of two independent experiments.

We next examined the contribution of TGF-β and RA to PP-DC-promoted IgA production by B2 cells by adding neutralizing anti-TGF-β monoclonal antibody (mAb) or the RARβ inhibitor LE135 to the co-culture. Both anti-TGF-β mAb and LE135 resulted in a partial inhibition (40–50%) of PP-DC-induced IgA production (Figure 3A). Interestingly, addition of LPS did not markedly alter the effect of either inhibitor (Figure 3B), indicating that LPS-mediated up-regulation of DC-promoted IgA production worked through similar mechanisms as those mediated by freshly isolated PP-DC. We next determined whether blockade of both TGF-β and RA could further decrease PP-DC mediated IgA production. The impact of adding both inhibitors resulted in a slight additive effect over the use of either inhibitor alone in the absence of LPS (p<0.05), whilst no significant difference was observed in the presence of LPS (Figure 3A&B). These data indicate that RA and TGF-β largely act through a common pathway to promote IgA. Indeed, Saurer et al. [36] recently demonstrated that RA can act on the DC itself to promote TGF-β production. Our findings are also in keeping with those of Mora et al. [25] who observed that RA could promote IgA production during in vitro B cell culture, but only when DC were included.

**Figure 4:**
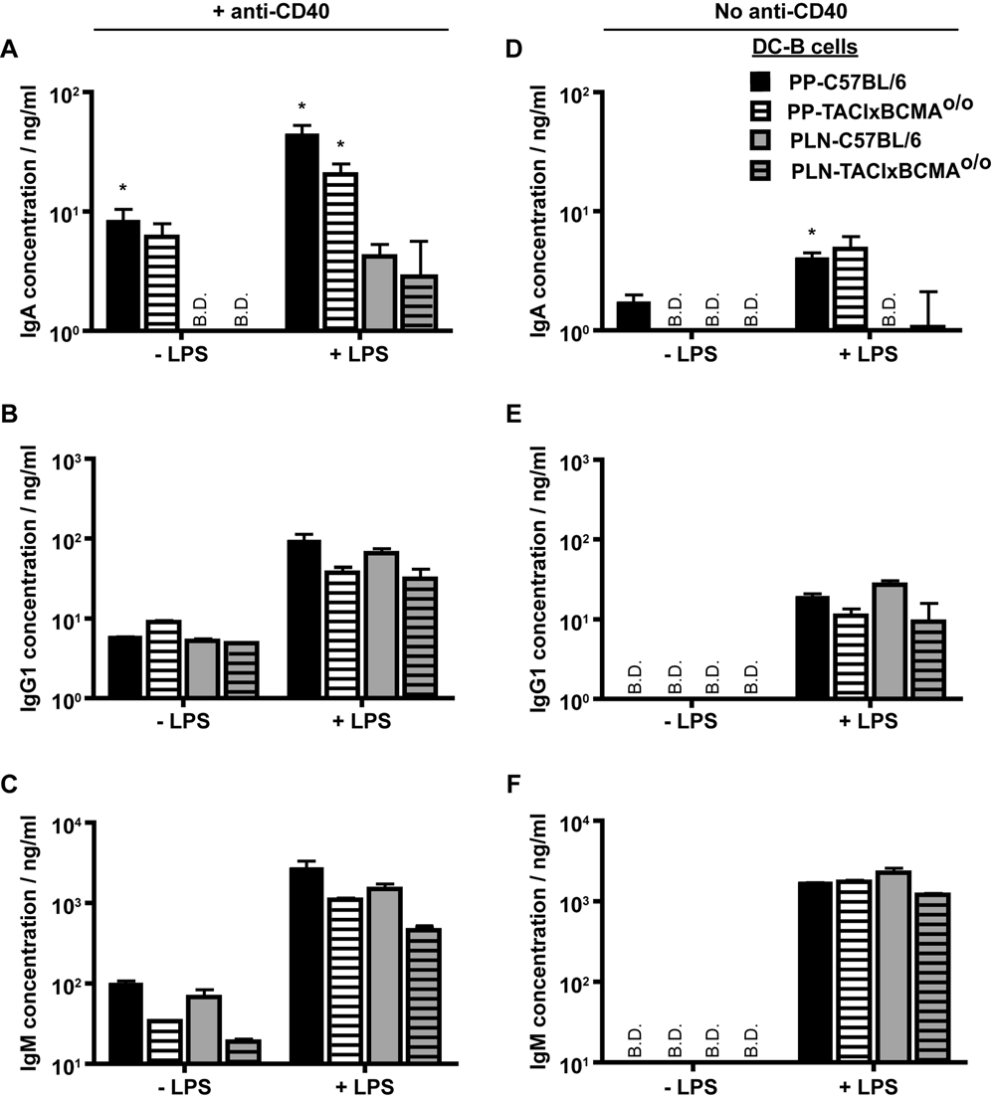
PP-DC promoted IgA production requires BCMA and TACI signaling in the absence of CD40- or LPS-mediated signals. Naïve splenic IgD^+^ B cells from C57BL/6 (plain bars) or TACIxBCMA^o/o^ mice (striped bars) were cultured together with CD11c^+^ DC from the PP (black and white bars) or PLN (grey bars). (A–C) Anti-CD40 mAb (2 µg/ml) was added to all cultures in the absence or presence of additional LPS (1 µg/ml) as indicated. (D–F) Cultures were performed in the absence of anti-CD40 mAb but contained LPS (1 µg/ml) where indicated. At the end of 7 days of culture, supernatants were collected and (A&D) IgA, (B&E) IgG1 and (C&F) IgM antibody concentrations determined by standard ELISA assay. Cultures were performed in triplicate and the mean±S.E.M. are shown. B.D. depicts those samples where the antibody concentration was below the detection limit of the ELISA assay. No IgA was detected in cultures containing DC alone. The data shown are from one experiment and are representative of two (D–F) and three (A–C) independent experiments.

The inability of both anti-TGF-β mAb and LE135 to completely inhibit IgA production indicated that other factors must contribute to the ability of PP-DC to promote IgA. We therefore investigated the contribution of BAFF and APRIL. As these molecules are known to promote IgA class switching through TACI and BCMA [37] we used naïve B2 cells isolated from mice deficient for these two molecules (TACIxBCMA^o/o^)[31] in our cultures. DC-promoted IgA levels were comparable between cultures of wildtype and TACIxBCMA^o/o^ B2 cells (Figure 4A), indicating that the absence of these molecules did not impact on IgA production. Nor was TACI and BCMA signaling required for anti-CD40 or LPS induced B2 cell IgG1 production (Figure 4B). Although the absence of TACI and BCMA signaling appeared to reduce anti-CD40 induced IgM production in the experiment shown (Figure 4C), IgM production did not differ between TACIxBCMA^o/o^ and C57BL/6 mice in two further experiments indicating that these molecules are unlikely to play an important role in IgM production (data not shown).

It remained possible that DC-secreted BAFF and APRIL could contribute to IgA production, but only in the absence of CD40 signaling. We therefore examined whether PP-DC could promote CD40-independent antibody production. IgM and IgG1 production was not observed in the absence of CD40 ligation regardless of the presence of PP- or PLN-DC, but was increased by LPS stimulation (Figure 4E&F). In contrast, a small (although not statistically significant) amount of CD40-independent IgA production could be detected in cultures containing PP-DC (Figure 4D). Importantly the actual level of IgA noted in the absence of CD40 ligation was minimal compared to that seen in the presence of anti-CD40 mAb, and was only observed in five out of a total of eight experiments. When CD40-independent IgA was noted, it was also observed to require TACI and BCMA signaling (Figure 4D). The addition of LPS co-stimulatory signals could overcome the requirement for TACI and BCMA and promoted increased CD40-independent IgA levels in cultures of both wildtype and TACIxBCMA^o/o^ B2 cells (Figure 4D). However, these levels were again very small.

### Intestinal bacteria contribute to the IgA-promoting phenotype of PP-DC

**Figure 5:**
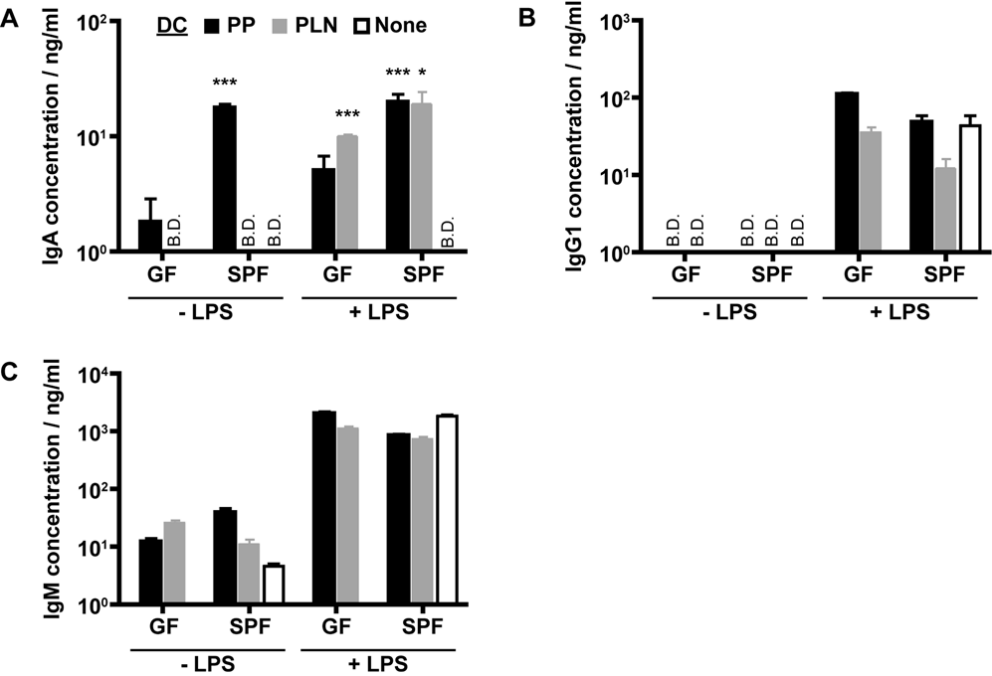
Intestinal commensal bacteria are partially responsible for the ability of PP-DC to promote IgA production. Naïve splenic IgD^+^ B cells from C57BL/6 mice were cultured alone (open bars) or together with CD11c^+^ DC from the PP (black bars) or PLN (grey bars). CD11c^+^ DC were isolated from mice maintained under SPF or GF conditions as indicated. Anti-CD40 mAb (5 µg/ml) was added to all cultures in the absence or presence of additional LPS (1 µg/ml) as indicated. At the end of 7 days of culture, supernatants were collected and (A) IgA, (B) IgG1 and (C) IgM antibody concentrations determined by standard ELISA assay. Cultures were performed in triplicate and the mean±S.E.M. are shown. B.D. depicts those samples where the antibody concentration was below the detection limit of the ELISA assay. No IgA was detected in control cultures containing DC alone. The data shown are from one experiment and are representative of three independent experiments.

Although numerous studies have now reported a role for GALT-DC in promoting mucosal lymphocyte differentiation, the factors responsible for imparting DC with this function remain largely unknown. Intestinal commensal bacteria are well known to impact on the organization and function of the immune system [38], and we observed that LPS could impart PLN-DC with an IgA promoting phenotype similar to that of PP-DC. These data led us to test the possibility that intestinal bacteria-derived factors may imprint PP-DC with an IgA-promoting phenotype. To investigate this possibility we isolated DC from GF or SPF housed mice, and cultured these cells together with naïve B2 cells isolated from SPF mice. In keeping with our earlier data DC from SPF mice were able to promote IgA production and this effect was most dramatic in the PP-DC population (Figure 5A). PP-DC isolated from GF mice exhibited a severely reduced capacity to promote IgA production as compared to SPF-DC (p = 0.0005). The inclusion of LPS in the GF DC cultures was able to largely overcome this defect and, as observed previously, also allowed PLN-DC to promote IgA production (Figure 5A). However, for both PLN- and PP-DC the ability of GF DC to promote IgA still remained reduced compared to SPF DC (p<0.05 for PP-DC). IgM and IgG1 production remained independent of the presence of DC, and the production of both antibodies was promoted by LPS (Figure 5B&C).

It remained possible that IgA production in cultures containing PP-DC from SPF, but not GF mice, could be accounted for by the presence of intestinal bacteria, or their fragments, within the SPF DC resulting in transfer of bacterial antigens to naïve B cells. However, we consider this possibility unlikely as further stimulation of B2 cells in GF PP-DC cultures by IgM cross-linking antibodies did not increase IgA levels, demonstrating that optimal BCR stimulation does not alter the attenuated capacity of GF PP-DC to promote IgA production (data not shown).

## Discussion

We have demonstrated that DC are required to promote IgA production by naïve B2 cells when these cells are stimulated in the absence of exogenous cytokines. By contrast, DC were not required for IgM or IgG1 production. This indicates that DC selectively promote IgA production, as opposed to simply impacting on B2 cell survival or transferring captured antigen. IgA secretion was most pronounced when the DC were isolated from the PP indicating that these DC exhibit a specialized phenotype that allows them to preferentially promote IgA production. Others have also reported that PP-DC selectively drive IgA production when added to T cell and B cell co-cultures [39]–[44]. Our data support these findings and further show that PP-DC can promote IgA production when T cell help is substituted by anti-CD40 mAb. These data indicate that DC can directly influence the IgA production by naïve B2 cells as opposed to simply promoting the secretion of cytokines by T helper cells.

These findings implied that the PP-DC represent a specialized subpopulation of cells, or that DC located within intestinal lymphoid tissues are conditioned by the local microenvironment to promote the production of mucosally relevant antibody isotypes. We therefore investigated the gene expression of factors known to be associated with IgA production in PP- and PLN-DC. PP-DC expressed increased levels of RALDH1, BAFF, APRIL, and VIP receptors. Inclusion of an RARβ inhibitor in our culture led to a partial reduction in IgA production and we concluded that RA may function to promote IgA production by stimulating the release of active TGF-β from PP-DC as inhibition of TGF-β also led to a partial reduction in IgA, whilst inhibition of both RA and TGF-β did not lead to an additive effect.

Since inhibition of both RA and TGF-β could not fully account for the ability of PP-DC to promote IgA production, we questioned whether BAFF and APRIL may play a role. For this purpose we used B2 cells lacking TACI and BCMA, but failed to observe a role for these molecules in CD40-dependent IgA production. As APRIL-TACI signaling has been widely implicated in T-independent IgA class switching [12] we next investigated a role for this pathway in absence of CD40. CD40-independent IgA production required intact TACI and BCMA signaling, however, DC-promoted IgA production by wildtype B2 cells was minimal in the absence of CD40-signaling. Moreover, the addition of LPS was able to overcome the requirement for BAFF and APRIL in this process. These data indicate that BAFF and APRIL are only necessary for IgA production by B2 cells in the absence of both CD40 and LPS co-stimulatory signals. Although it has been postulated that APRIL stimulation of B2 cells may contribute to CD40-independent IgA production [12], we believe that IgA production in mice lacking T cells [15], [45], or CD40 [46], is more likely to be derived from B1 cells.

The ability of the microenvironment to impact on immune cell function, including that of DC, is well recognized—yet little is known about the exact mechanisms by which this process occurs. Mice housed under GF conditions lack significant IgA [38] and recolonization with a normal bacterial flora [47], or even mono-association with segmented filamentous bacteria [48], [49] can restore IgA levels. We therefore compared the ability of DC isolated from mice housed under SPF or GF conditions to promote IgA production in a co-culture using SPF B2 cells. PP-DC isolated from GF mice were significantly less potent in promoting IgA production indicating that intestinal commensal bacteria can impact directly on DC function.

Intriguingly, Tezuka et al. [34] recently reported that iNOS is required for IgA production and that iNOS-positive DC are more potent at promoting T-dependent IgA production than their negative counterparts through a TGF-β-mediated mechanism. Toll-like receptor ligands derived from intestinal bacteria, such as LPS, are well known to promote iNOS expression [50] indicating that these microorganisms may impact directly on the ability of DC to promote IgA by regulating iNOS. Indeed, the finding that LPS was able to restore IgA production in cultures containing DC from GF mice supports this hypothesis. In further confirmation of this hypothesis we observed increased expression of iNOS in PP-DC versus PLN-DC. Thus, we believe that commensal intestinal bacteria are likely to signal through pattern-recognition receptors such as Toll-like receptors to mediate iNOS upregulation on local PP-DC, which in turn results in increased capacity of these cells to promote IgA switching of antigen-specific B2 cells in a CD40-dependent manner. This process most likely occurs through a TGF-β-dependent pathway which also requires the local production of RA, although other mechanisms are also likely to be involved. In addition, these PP-DC produce increased amounts of BAFF and APRIL which function to promote the production of CD40-independent IgA, although this may largely derive from B1 cells.

Even though commensal intestinal bacteria clearly impacted on DC function, PP-DC isolated from GF mice were still able to promote a low level of IgA secretion indicating that other factors contributed to their conditioning *in vivo*. IEC are known to secrete numerous chemokines and cytokines which impact on immune function [51]–[53]. Co-culture of epithelial cells together with DC has been reported to modulate DC function [54], [55] indicating that these cells may account for the residual activity of PP-DC isolated from GF mice in the promotion of IgA production. Another possible mechanism by which PP-DC may be conditioned to promote IgA production is through stimulation by neuroendocrine peptides present within the intestinal microenvironment. VIP is present in high concentration within the intestine and has been reported to promote IgA synthesis [32], [33] and to modulate DC function [56], [57]. We have shown that DC isolated from the PP express increased levels of VIP receptors, VPAC1 and VPAC2, as compared to peripheral DC, indicating that these DC are susceptible to modulation by VIP and raising the possibility that VIP stimulation may contribute to the ability of these cells to promote IgA production. However, initial attempts in our laboratory to condition peripheral DC through the addition of VIP have been unsuccessful. We consider it likely that such DC are unresponsive to VIP due to the reduced expression of VIP receptors on peripheral DC and that additional, as yet unidentified, factors may be required to enhance their VPAC1 and VPAC2 expression.

In summary, we have demonstrated that DC are required for IgA plasma cell differentiation *in vitro*, with PP-DC exhibiting the most potent IgA promoting activity. Factors derived from commensal bacteria, that are unique to the intestinal microenvironment, were found to modulate DC function such that these cells preferentially promote the production of the IgA antibody isotype. These findings provide further evidence that intestinal immune response is highly specialized for the purpose of providing protective immunity whilst preserving intestinal integrity, and that the dominant production of IgA antibodies forms an integral part of this process. They additionally highlight the central role DC play in providing a bridge between bacterial and tissue-specific signals and adaptive immunity.
